# The FDA Reclassification of Cervical Pedicle and Lateral Mass Screws: A Case Study in Regulatory History

**DOI:** 10.1007/s43441-024-00654-1

**Published:** 2024-04-15

**Authors:** Jonathan H. Sussman, Ahmed Albayar, Anissa Saylany, Bhargavi R. Budihal, Dominic Romeo, Jason Xu, Joshua Rosenow, Robert F. Heary, William C. Welch

**Affiliations:** 1 Department of Neurosurgery, Perelman School of Medicine, 3400 Civic Center Blvd, Floor 15, 19104 Philadelphia, PA USA; 2 Medical Scientist Training Program, University of Pennsylvania, Philadelphia, PA USA; 3 Division of Plastic, Reconstructive and Oral Surgery, Children’s Hospital of Philadelphia, Pennsylvania, USA; 4 Department of Neurological Surgery, Northwestern University Feinberg School of Medicine, Chicago, Illinois USA; 5 Division of Neurological Surgery, Mountainside Medical Center, Hackensack Meridian School of Medicine, Montclair, New Jersey USA

**Keywords:** Cervical screws, Device classification, FDA regulation, Food, drug, and cosmetic act, Lateral mass screws, Pedicle screw systems

## Abstract

The classification of medical devices by the Food and Drug Administration (FDA) involves rigorous scrutiny from specialized panels that designate devices as Class I, II, or III depending on their levels of relative risk to patient health. Posterior rigid pedicle screw systems were first classified by the FDA in 1984 and have since revolutionized the treatment of many spine pathologies. Despite this early classification by the FDA, posterior cervical pedicle and lateral mass screws were not reclassified from unclassified to Class III and then to Class II until 2019, nearly 35 years after their initial classification. This reclassification process involved a decades-long interplay between the FDA, formal panels, manufacturers, academic leaders, practicing physicians, and patients. It was delayed by lawsuits and a paucity of data demonstrating the ability to improve outcomes for cervical spinal pathologies. The off-label use of thoracolumbar pedicle screw rigid fixation systems by early adopters assisted manufacturers and professional organizations in providing the necessary data for the reclassification process. This case study highlights the collaboration between physicians and professional organizations in facilitating FDA reclassification and underscores changes to the current classification process that could avoid the prolonged dichotomy between common medical practice and FDA guidelines.

## Introduction: Current FDA Device Classification Process

**Fig. 1:**
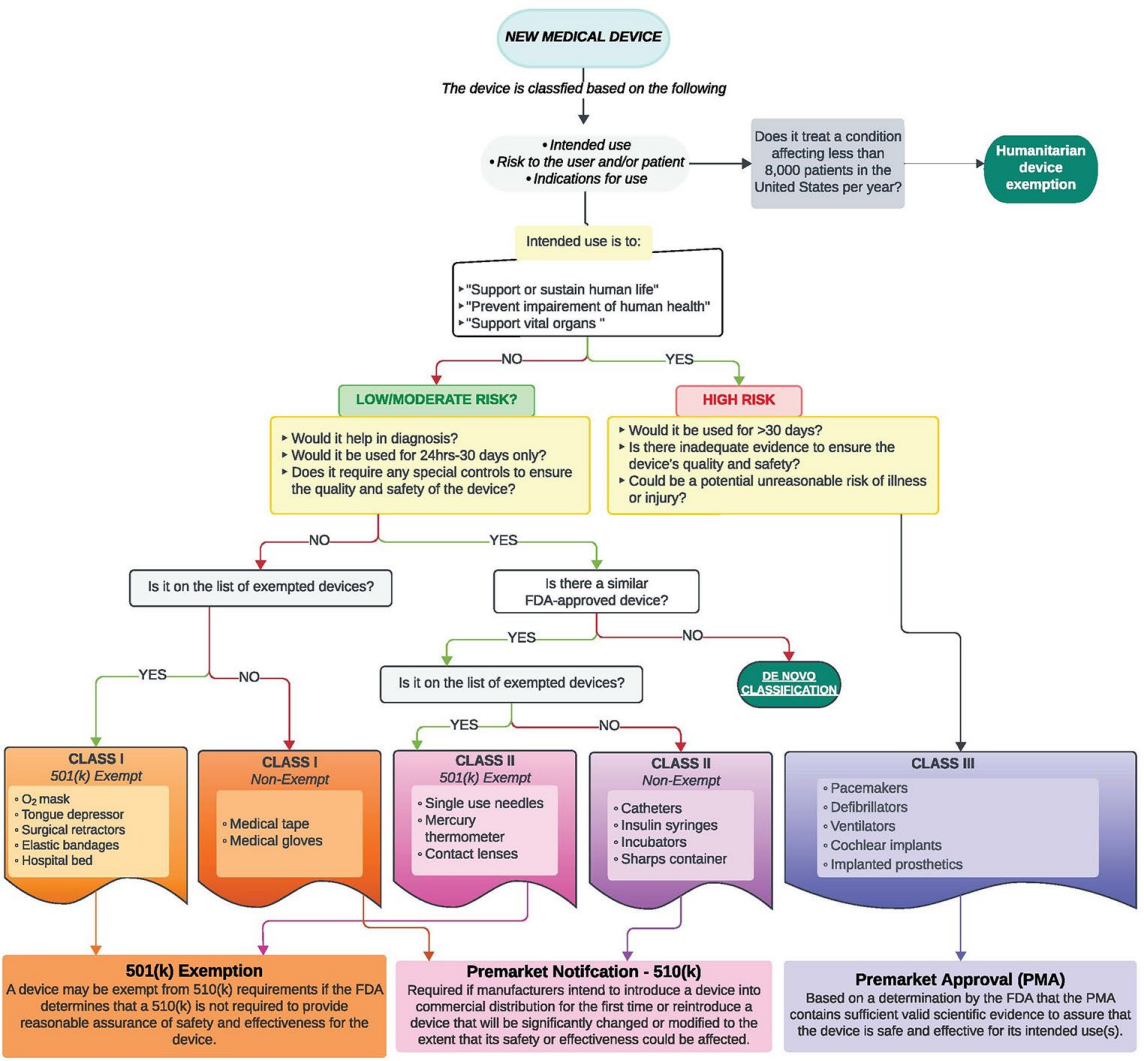
Flowchart of typical device classification pathways for a new medical device

The Food and Drug Administration (FDA) is responsible for classifying the safety of more than 6,500 different medical devices [1]. This is achieved through an often lengthy process that involves specialty panels within the FDA that analyze data on these devices. The Federal Food, Drug, and Cosmetic Act (FD&C Act) mandates that the FDA classify medical devices into one of three classes (Class I-III) considering the intended use of each device and the data surrounding the indications for its use (Fig. 1) [2]. The classification involves an evaluation of the “acceptable risk” for patient health. Each class designates a distinct set of regulatory controls deemed necessary to ensure safe and effective use, including special labeling, design specification, performance standards, and guidance documents [1, 2]. 

This process of device classification is dynamic and continuously evolves as physicians, researchers, and device manufacturers collect more data about the safety and efficacy of devices. Importantly, there is often a significant dichotomy between common medical practice and FDA guidelines, as physicians frequently use devices for indications other than those for which the device is specifically approved to be marketed, highlighted here through the history of cervical screws. While physicians are permitted to use devices off-label, there is value in aligning FDA classifications with clinical practice. Beyond issues of medical liability, appropriate device classifications improve reimbursement and insurance coverage for patients, making devices more accessible. In addition, accurate classifications facilitate the development and marketing of future devices, which can gain approval based on similarities to existing products. Physicians play an active role in this process through routine clinical practice and the curation of clinical data. Therefore, it is beneficial for practicing physicians to be familiar with the complexities of this process and recognize the importance of their collective roles in device regulation.

The three device classes are as follows: Class I devices are those for which general controls of the FD&C Act can ensure safety and efficacy. These controls include protections against the misbranding of devices, oversight of device registrations and records, and general reports on devices. Class I controls apply to all medical devices unless they receive an exemption upon classification. Examples include adhesive bandages and stethoscopes.

Class II devices are considered moderate-risk devices that require both general and special controls. The special controls are device-specific and include oversight of performance standards, post-market surveillance, patient registries, special labeling, premarket data requirements, and guidelines. These devices span a broad range from simple devices such as surgical masks and electric wheelchairs to more complex devices such as blood transfusion devices and electric wheelchairs.

Class III devices are considered high-risk devices that “support or sustain human life” and are therefore subject to the most stringent regulatory controls, requiring both general controls and premarket approval (PMA) [2]. Gaining PMA requires an application that functions as a private license to market a particular device. If a Class III device fails to meet PMA requirements, Sect. 501(f) of the FD&C Act prohibits it from being marketed for clinical use. This category includes most implantable devices such as implantable neurostimulators and breast implants. Overall, 35% of all devices are Class I, 53% are Class II, 9% are Class III, and 3% are unclassified [1–3]. 

## 1976 Medical Devices Amendment

On May 28, 1976, the FDA instituted a set of Medical Device Amendments to the Federal Food, Drug, and Cosmetic Act (FD&C Act), Sect. 513, to establish the medical device classification system. Section 210(h) of this Act defined a “medical device” as “any product that does not achieve its purposes by chemical action or metabolization.” [4] Medical devices seeking FDA classification are divided into either “pre-amendment” or “post-amendment” categories, depending on whether the device was in commercial distribution before or after May 28, 1976. This determination is made through the premarket notification procedure outlined in Sect. 510(k) of the FD&C Act, and if these devices came to market after May 28, 1976, then these devices are automatically classified as Class III, thus requiring PMA, with the possibility of later reclassification as either Class III (requiring 510(k)) or Class II after a full FDA review is completed [4]. 

Pre-amendment devices are classified according to the following process. First, the FDA receives recommendations from an FDA-instituted device classification panel. Then, the FDA publishes the panel’s recommendations with their proposed regulations for public comments. Lastly, the FDA publishes a final regulation designation that classifies the device through an administrative order [5]. 

Post-amendment devices are automatically classified as Class III without any FDA rulemaking proceedings. These devices remain Class III unless the device is reclassified as Class I or II unless the FDA finds the device equivalent to a device not requiring PMA [5]. The later reclassification of a medical device by administrative order can take place based on the re-evaluation of data available at the time of initial classification or additional publicly available data that offers new insight into device safety and efficacy [6]. 

## The Role of the Rigid Pedicle Screw Systems in Spine Surgery

Medical issues related to the spine such as chronic back pain are exceedingly common. Approximately 900,000 adults in the U.S. undergo spine surgery annually, most commonly receiving spinal decompression or fusion surgeries, which involve some form of spinal fixation [7]. Spinal fixation is a common technique used for many conditions, including spinal fractures, reconstruction after tumor removal, scoliosis, spondylolisthesis, disk disease, and degenerative arthritis [8]. This improves spinal stability, allowing for better healing, greater mobility, and decreased pain. In current medical practice, there is a wide range of instrumentation and techniques, but can be divided generally based on the anatomic location in which the systems are used, from the neck to the lower back: occipitocervical junction, lower cervical spine, thoracic and lumbar spine, and lumbosacral spine [8]. Additionally, these systems are further classified by the anatomic portion of the spinal vertebrae in which the screws are inserted, often the lateral mass or the pedicle.

Thoracolumbar pedicle screw systems were first proposed by Raymond Roy-Camille of France in the early 1960s to treat spinal fractures, and they were subsequently marketed in the United States [9]. In the 1970s, Roy-Camille later introduced posterior cervical lateral mass screw fixation systems that were quickly adopted by spine surgeons globally for spinal stabilization [10]. The development of cervical pedicle screw fixation systems followed in the late 1980s and early 1990s [11]. The combination of these advancements ultimately revolutionized the modern treatment of cervical spinal disorders [11]. Collectively, these systems mechanically align segments of the spine using a metal structure in order to provide three-column support of the spinal vertebrae, thus facilitating bony fusion [12]. These devices allow for rigid fixation even in the face of aggressive bony decompression and offer more effective resistance to lateral bending and axial rotation compared to previous devices [13–15]. This has dramatically improved the postoperative course of patients undergoing posterior cervical fusions and has contributed to the increased utilization of posterior cervical fusions to treat cervical spine pathologies [16, 17]. Despite the advantages of the posterior cervical pedicle and lateral mass screws, and their routine use in clinical practice, it was not until 2019 that the FDA reclassified them from an unclassified category to Class II devices [18]. 

## Early Classification of Pedicle Screw Spinal Systems in the 1980s and 1990s

The first premarket notification 510(k) submission to the FDA for spinal device systems used for attachment to the spine via lumbar pedicles was in 1984 [6]. At the time, the FDA decided that the pedicle devices were not substantially equivalent to other approved devices and expressed concern about unique risks such as potential neurological deficits due to imprecise screw placement and screw failure. These devices are placed in close proximity to the spinal cord, and possible misalignment or breakdown over time could, in theory, lead to irreversible spinal cord damage and paralysis. Thus, pedicle screw systems were made Class III devices under Sect. 513(f) of the FD&C Act [6]. Despite the numerous benefits of these systems, given the theoretical possible for life-threatening adverse events, the most stringent classification was necessary to ensure rigorous manufacturing oversight. By 1985, clinical investigations of pedicle screw spinal systems under investigational device exemption (IDE) protocols began, allowing pedicle screw spinal systems to be used in clinical studies to collect data to support a PMA application, a humanitarian device exemption (HDE), or a premarket notification 510(k) submission to the FDA [6]. 

By 1992, the FDA acknowledged that the use of pedicle screw spine systems outside of approved IDE studies was widespread in the surgical community to the point that they were considered “standard of care.” [6] Then, in February 1993, the FDA called for the submission of all available data on pedicle screw systems. This prompted the formation of the Spinal Implant Manufacturers Group (SIMG) which consisted of representatives from the American Academy of Orthopedic Surgeons (AAOS), the Scoliosis Research Society (SRS), the North American Spine Society (NASS), the American Association of Neurological Surgeons (AANS), the Congress of Neurological Surgeons (CNS), and 25 spinal implant manufacturers. The SIMG scientific committee, with FDA support, conducted a nationwide historical cohort study of pedicle screw fixation in thoracic, lumbar, and sacral spine surgeries [6, 19]. On August 20, 1993, the FDA convened an advisory panel to review the IDE data, mechanical testing data, and presentations by experts. The FDA advisory panel concluded that pedicle screw spinal systems were reasonably safe spinal fusion adjuncts; however, they held that more clinical information was needed for the establishment of the necessary regulatory controls prior to their approval [6]. 

In July 1994, the FDA advisory panel evaluated the SIMG cohort study and two meta-analyses of the literature pertaining to the clinical performance of pedicle screw spinal devices [6]. The panel also heard testimonies of patients with pedicle screw instrumentation, litigation attorneys with lawsuits against spine implant manufacturers, and spine surgeons. The panel concluded that pedicle screw use in spinal instrumentation systems, specifically in the thoracolumbosacral spinal systems, was appropriate for the treatment of spinal instabilities including trauma, deformity, tumor reconstruction, and spondylolisthesis. However, they expressed concern about their use for degenerative disc disease due to a dearth of satisfactory data on the topic. Ultimately, the panel recommended that the FDA reclassify generic pedicle screw spinal systems from Class III to Class II for the treatment of degenerative spondylolisthesis and spinal trauma. At this point, the FDA had not yet directly addressed pedicle screw or lateral mass screw utilization in the cervical spine in panels or formal proposals, and cervical screws remained Class III devices [6]. 

In 1995, the FDA advisory panel formally recommended reclassifying pedicle screw spinal systems used for autogenous bone graft fusion in severe L5-S1 spondylolisthesis, with device removal post-fusion, as Class II [19]. In addition, the thoracolumbosacral pedicle screw systems were recommended for use in spinal trauma and formally proposed for the treatment of acute and chronic spinal instability and deformity. On July 17, 1998, the FDA issued a final ruling to classify pedicle screw spinal systems as Class II devices when used specifically to provide immobilization and stabilization of thoracic, lumbar, and sacral spinal segments as an adjunct to fusion in the treatment of degenerative spondylolistheses. All other indications were deemed Class III, and thus still required a PMA for advertisement of use in those indications [19]. In this same ruling, cervical pedicle screws were excluded from updated classification and were still considered post-amendment Class III devices with the FDA, citing insufficient safety and efficacy evidence [19]. 

## Petitions and Panels for Reclassification of Cervical Pedicle Screw Spinal Systems

On May 22, 2001, the FDA issued a technical amendment to its 1998 ruling. In this amendment, the FDA noted that lateral mass and pedicle screw systems in the cervical spine were in use before 1976 and should instead be considered pre-amendment Class III devices instead of post-amendment Class III devices. This ruling subjected cervical spine pedicle screw systems to specific regulations such as requiring 510(k) premarket notifications [20]. 

Eight years later, on April 9, 2009, the FDA published an order under Sect. 515(i) of the FD&C Act (21 U.S.C. 360i) calling for additional information on the remaining Class III 510(k) pre-amendment devices including the thoracolumbosacral pedicle screw spinal systems [21]. However, the classification of posterior cervical screws was deferred due to a lack of prior input from the FDA advisory panel. Responses to this 515(i) order concerning thoracolumbosacral spinal systems unanimously supported Class II designation with assurances provided by special controls such as labeling, biocompatibility, sterility, and mechanical testing [21]. This was an important advancement because Class II devices are deemed lower risk than Class III devices, and therefore, can be more readily used in patient care. Additionally, designation from Class III to Class II has the potential to improve insurance coverage of certain procedures, making them more accessible to patients.

In May 2010, the Orthopedic Surgical Manufacturers Association (OSMA) responded to the 515(i) order with a petition declaring that posterior screws, particularly pedicle screws, should be deemed Class II devices for use in degenerative disc disease, pediatrics, and the posterior cervical spine [22]. In November 2010, the AANS and CNS also submitted comments in support of the OSMA petition. In this letter, the organizations pointed to the challenges of the growing off-label use of cervical pedicle screw spinal devices, including medical liability for surgeons, inappropriate denial of insurance coverage, and published lack of trust in the validity of use and cost [22]. The inclusion of data regarding posterior cervical screws from OSMA and others was outside the scope of the original 2009 FDA “call for information.” The topic of posterior cervical pedicle screw systems had yet to be formally addressed in a classification panel meeting and it was required that a panel be convened per Sect. 513(b) of the FD&C Act [23]. Therefore, the FDA requested that OSMA prepare and submit a separate petition for classification.

In November 2011, OSMA submitted a petition for lateral mass and pedicle screw spinal systems to be moved to a Class II designation. In this petition, OSMA sought to reclassify cervical pedicle screw systems at levels C1 to T3 as Class II due to the ability of the general and special controls to provide a reasonable assurance of safety and effectiveness [22]. The petition included a literature search of cervical spine pedicle screw systems in practice (including data from 1999 to 2009) and identified that such systems offered patients improved or preserved neurological function, improvements in pain/discomfort, and higher fusion rates when compared to already Class II-designated cervical spinal sublaminar hooks, sublaminar cables, and interspinous process wiring [22]. 

On August 28, 2012, a panel from the AANS/CNS submitted another letter with comments regarding lateral mass and pedicle screw spinal system classification in response to the FDA Orthopedic and Rehabilitation Devices Panel meeting of the medical devices advisory committee (MDAC) [24]. This letter emphasized the anatomical and biomechanical advantages of posterior cervical screws in comparison to FDA-approved devices and pointed to many studies that supported their safety and utility. They emphasized that pedicle screws used in the posterior cervical spine were widely considered standard of care and called for Class II designation for posterior cervical lateral mass and pedicle screw systems [24]. 

In September 2012, the FDA consulted with the Orthopedic and Rehabilitation Devices Panel FDA advisory committee regarding posterior cervical pedicle and lateral mass screw spinal systems. Based on their expertise and historical data, the panel noted effective posterior cervical pedicle and lateral mass screw spinal systems were in use as adjuncts to fusion for acute and chronic instabilities of the cervical spine and craniocervical junction [23]. The panel did express concerns regarding a lack of long-term data and the need for additional data on specific screw types. At the meeting’s conclusion, an unofficial vote by the panel was in favor of posterior cervical pedicle and lateral mass screw spinal systems to be designated as Class II devices [25]. In May 2013, the same panel convened and recommended that rigid pedicle screw systems have an additional Class II designation when used in the thoracic, lumbar, and sacral spines as adjuncts to fusion for degenerative disc disease and spondylolisthesis (other than either severe spondylolisthesis grade 3 or 4 at L5-S1 or degenerative spondylolisthesis with objective evidence of neurologic impairment). In support of their recommendation, the panel included a summary of safety and effectiveness information, a literature review, and a review of adverse event reports from the Manufacturer and User Facility Devices Experience (MAUDE) database [26]. 

## Final Classification Proposals and Rulings

In November 2014, the FDA proposed the reclassification of rigid pedicle screw systems based on new information. At the time of the proposal, rigid thoracolumbosacral pedicle screw systems were still Class III pre-amendment devices for some spinal pathologies. The proposed administrative order, as governed by Sect. 513(e) of the FD&C Act, would reclassify rigid pedicle screw systems when intended to provide immobilization and stabilization of spinal segments in the thoracic, lumbar, and sacral spines as adjuncts to fusions in the treatment of degenerative disc disease and spondylolisthesis (with the previously noted exception) from Class III to Class II [27]. 

On March 10, 2016, the FDA proposed the classification of posterior cervical screw systems—including lateral mass and pedicle screws—as Class II. Posterior cervical screw systems were identified as those intended to provide immobilization and stabilization of spinal segments in patients as an adjunct to fusion for acute and chronic instabilities involving the cervical spine. The proposal claimed that while cervical pedicle screw systems are similar in design and operation to thoracolumbosacral pedicle screw spinal systems, they differ with respect to anatomical use and clinical indications. Therefore, they had to be considered separately in terms of classification and determination of necessary controls [28]. 

The FDA also conducted a literature review that included data until July 2012 reviewing published clinical studies as well as a review of adverse events in the MAUDE Database. This review, along with advice from the September 2012 Panel, suggested that Class II controls would be sufficient to ensure the safety and efficacy of posterior cervical screw systems. Additionally, an economic analysis urged that the reclassification of posterior cervical screw systems to Class II would not have a negative economic impact on various small entities as only five registered establishments at the time would have been affected by the proposed rule [23]. 

Following the March 2016 proposal, on December 30, 2016, the FDA put forth a final order to classify thoracic, lumbar, and sacral rigid pedicle screw systems as adjuncts to fusions in the treatment of degenerative disc disease and spondylolisthesis (with the previously noted exception) as Class II devices [4]. Lastly, on April 1, 2019, the FDA issued a final rule to classify posterior cervical screw systems as Class II, with a continued requirement of premarket notification 510(k) to ensure reasonable safety and effectiveness of the device [18, 28]. 

## Improving Device Classification Through Data-Driven Collaboration

This case study of the FDA classification of cervical pedicle and lateral mass screws presents a comprehensive history of a significant medical device (summarized in Table 1) and illustrates the complexities of the FDA regulatory process. This process has involved a decades-long interplay between the FDA, formal panels, manufacturers, academic leaders, and practicing physicians, and only recently concluding.

**Table 1:** Timeline of FDA classifications of the spinal pedicle screw rigid systems highlighting the separation between thoracolumbosacral fusions and their use for posterior cervical fusions

Mid 1992	The FDA acknowledged the off-label use of pedicle screws.
February 1993	The FDA called for submission of pedicle screw system-related data.
August 1993	The FDA advisory panel found pedicle screw spinal systems to be reasonably safe spinal fusion adjuncts; however, they desired additional data prior to establishing regulatory controls around their use.
July 1994	The FDA advisory panel concluded that the use of pedicle screw spinal systems in the thoracolumbosacral spine was appropriate for treating spinal instabilities, but they expressed concern about the use for degenerative disc disease. Ultimately the panel recommended that the FDA reclassify generic pedicle screw spinal systems from Class III to Class II for treating degenerative spondylolisthesis and spinal trauma. At this point, the FDA had not formally addressed the use of the pedicle screw system in the cervical spine.
October 1995	The FDA formally proposed that pedicle screw spinal systems used for autogenous bone graft fusions in patients with severe L5-S1 spondylolisthesis with device removal post-fusion be classified to Class II. The FDA also recommended post-amendment thoracolumbosacral pedicle screw spinal systems intended for use in spinal trauma and degenerative spondylolisthesis be reclassified from Class III to Class II. Additionally, they proposed expanding the intended use of pedicle screw spinal systems to include immobilization and stabilization of spinal segments as an adjunct to fusion for the treatment of acute and chronic instabilities and deformities.
July 1998	The FDA issued a rule that set forth stipulations to split pedicle screw spinal systems into different classification categories. They classified pedicle screw spinal systems as Class II devices when used to provide immobilization and stabilization of thoracic, lumbar, and sacral spinal segments as an adjunct to fusion in the treatment of degenerative spondylolisthesis with objective evidence of neurologic impairment, fracture, dislocation, scoliosis, kyphosis, tumor, and/or failed prior fusion. All other indications were deemed Class III, requiring a PMA for use.
May 2001	The FDA issued a technical amendment to their 1998 ruling that included an additional use for Class II designation: pedicle screw spinal systems in the treatment of severe spondylolisthesis (grades 3 and 4) at the L5-S1 level as an adjunct to fusion.
April 2009	The FDA called for information on the remaining Class III 510(k) pre-amendment devices, of which pedicle screw spinal systems for use in the thoracic, lumbar, and sacral spine as an adjunct to fusion for treatment of degenerative disc disease and types of spondylolisthesis for which the classification process had yet to be finalized.
September 2012	The FDA consulted the Orthopedic and Rehab Devices Panel FDA advisory committee regarding cervical screw systems. The panel members cited their knowledge and clinical experiences with cervical screw systems, and the historical data for the safety and efficacy of such devices. At the meeting’s conclusion, an unofficial vote favored the designation of posterior cervical screws as Class II devices.
November 2014	The FDA proposed reclassifying rigid pedicle screw systems based on “new information.” At this time, rigid thoracolumbosacral pedicle screw systems were largely deemed Class III devices.
March 2016	The FDA proposed the classification of posterior cervical lateral mass and pedicle screw systems as Class II. Posterior cervical screw systems were identified as those that provided immobilization and stabilization of spinal segments in patients as an adjunct to fusion for instabilities of the cervical spine, craniocervical junction, and/or cervicothoracic junction. The proposal claimed that given cervical pedicle screw systems differed in indication and anatomical location to thoracolumbosacral pedicle screw spinal systems, they had to be classified separately from each other.
December 2016	The FDA suggested classifying thoracic, lumbar, and sacral rigid pedicle screw systems adjuncts to fusions in treating degenerative disc disease and spondylolisthesis as Class II devices. Posterior cervical screws remained unclassified.
April 2019	The FDA issued a final rule to reclassify posterior cervical screw systems as Class II, with a continued requirement of premarket notification 510(k) to ensure reasonable safety and effectiveness of the device.

Notably, cervical screws are an unlikely member of the Class II family, given that they are surgically implanted, which typically requires Class III controls, and may not seem to be in the same risk category as other Class II devices such as surgical masks and electric wheelchairs. The history of cervical screws reveals that our understanding of medical risk is continuously developing and evolves with data that is collected across thousands of patients over decades, with this acquisition of knowledge informing the classification of medical devices. While certain life-sustaining devices such as implantable pacemakers will likely remain as Class III due to their need for the most stringent safety controls, this case study suggests the possibility that other current Class III implantable devices may be reclassified to Class II, and that perhaps simple Class II devices may eventually be reclassified to Class I. Doing so would decrease regulatory costs, promote their use in clinical practice, and improve insurance coverage, ultimately rendering devices more accessible to a broader patient population and improving their care.

Looking back on this tumultuous process, this review elucidates specific ways in which healthcare providers, manufacturers, and regulators can make the classification process more effective. Primarily, these events demonstrate that the reclassification process will not occur spontaneously and requires the active engagement and collaboration between physicians, clinical researchers, manufacturers, professional societies, and the FDA to provide and interpret safety and efficacy data. The decades-long debates about the safety of cervical pedicle and lateral mass screws were driven by a lack of definitive and reliable data. Specifically, although these devices were used routinely in clinical practice for decades, the data on patient safety and efficacy was not sufficiently generalizable to the broad population of patients who may be treated by these medical devices. This requires that data be collected and analyzed to understand the effects of age, genetic background, comorbidities, and social factors on the safety and efficacy of medical treatments. However, healthcare providers and clinical researchers must take a proactive initiative to curate this information and conduct appropriate and rigorous analyses demonstrating that their conclusions can be generalized. Additionally, studies conducted in different medical centers may not be readily comparable due to discrepancies in patient populations and medical practices. Integrating disparate studies is challenging, and future regulatory efforts would benefit from more robust meta-analyses that curate larger amounts of clinical data to synthesize stronger conclusions.

Moreover, it is critical that this data is cogently presented to the FDA. The FDA has no direct interest in down classifying devices because its primary role is in keeping patients safe. Therefore, without the active engagement of physicians– as facilitated and promoted by professional organizations– the reclassification presented here would not have taken place. Although the process was lengthy, the FDA indeed served its intended purpose in fairly reviewing the data to presented to it and remaining cautious in loosening regulations. Physicians ultimately guided this endeavor and should recognize the invaluable role they play in shaping the process of device classification and enabling the adoption of new technologies.

Finally, the FDA has an obligation to facilitate this collaboration through improved transparency and data sharing. Although FDA regulatory meetings are often public, and transcripts are released, the exact data and analysis that guides these decisions are rarely compiled and nearly impossible to locate. Furthermore, it is unclear what precisely dictated the final votes of the panel members in this case. How the data was presented at the meeting and interpreted by the panel could lead to poor decisions with little corrective mechanism. It would be beneficial for the FDA to provide a comprehensive and quantitative overview of the relevant data presented and the interpretations by panel members. This would enable the broader academic community to critique the methods and conclusions in the same way that peer-reviewed studies are ultimately scrutinized after publication. By facilitating a more active and productive collaboration with physicians and manufacturers, the FDA likely could have significantly expedited this reclassification without compromising patient safety.

Overall, the regulatory history of cervical pedicle and lateral mass screws demonstrates how physicians and device manufacturers can help to reduce the frequent dichotomy between typical medical practice and FDA guidelines and facilitate classifications that most accurately reflect the current regulatory needs for common medical devices.

## Data Availability

No datasets were generated or analysed during the current study.
